# Sex-Specific Associations of Cardiovascular Risk Factors and Biomarkers With Incident Heart Failure

**DOI:** 10.1016/j.jacc.2020.07.044

**Published:** 2020-09-22

**Authors:** Navin Suthahar, Emily S. Lau, Michael J. Blaha, Samantha M. Paniagua, Martin G. Larson, Bruce M. Psaty, Emelia J. Benjamin, Matthew A. Allison, Traci M. Bartz, James L. Januzzi, Daniel Levy, Laura M.G. Meems, Stephan J.L. Bakker, Joao A.C. Lima, Mary Cushman, Douglas S. Lee, Thomas J. Wang, Christopher R. deFilippi, David M. Herrington, Matthew Nayor, Ramachandran S. Vasan, Julius M. Gardin, Jorge R. Kizer, Alain G. Bertoni, Norrina B. Allen, Ron T. Gansevoort, Sanjiv J. Shah, John S. Gottdiener, Jennifer E. Ho, Rudolf A. de Boer

**Affiliations:** a Department of Cardiology, University of Groningen, University Medical Center Groningen, Groningen, the Netherlands; b Cardiology Division, Department of Medicine, Massachusetts General Hospital and Harvard Medical School, Boston, Massachusetts; c Ciccarone Center for the Prevention of Heart Disease, The Johns Hopkins University, Baltimore, Maryland; d Department of Biostatistics, Boston University School of Public Health, Boston, Massachusetts; e Boston University School of Medicine and School of Public Health, and NHLBI and Boston University’s Framingham Heart Study, Framingham, Massachusetts; f Departments of Medicine, Epidemiology and Health Services, University of Washington, and Kaiser Permanente Washington Health Research Institute, Seattle, Washington; g Department of Family Medicine and Public Health, University of California, San Diego, La Jolla, California; h Department of Biostatistics, University of Washington, Seattle, Washington; i Center for Population Studies, National Heart, Lung, and Blood Institute, Bethesda, Maryland; j Department of Internal Medicine, Division of Nephrology, University of Groningen, University Medical Center Groningen, Groningen, the Netherlands; k Department of Medicine, Johns Hopkins Medical Institutions, and Department of Cardiology, Heart and Vascular Institute, The Johns Hopkins University, Baltimore, Maryland; l Department of Medicine and Pathology & Laboratory Medicine, University of Vermont Larner College of Medicine, Burlington, Vermont; m Institute for Clinical Evaluative Sciences, Toronto, Ontario, Canada; n University of Toronto, Toronto, Ontario, Canada; o Department of Internal Medicine, UT Southwestern Medical Center, Dallas, Texas; p Inova Heart and Vascular Institute, Falls Church, Virginia; q Section on Cardiovascular Medicine, Wake Forest School of Medicine, Winston-Salem, North Carolina; r Division of Cardiology, Department of Medicine, Rutgers New Jersey Medical School, Newark, New Jersey; s Departments of Medicine, Epidemiology and Biostatistics, San Francisco Veterans Affairs Health Care System and University of California-San Francisco, San Francisco, California; t Division of Public Health Sciences, Department of Epidemiology and Prevention, Wake Forest School of Medicine, Winston-Salem, North Carolina; u Department of Preventive Medicine, Northwestern University Feinberg School of Medicine, Chicago, Illinois; v Division of Cardiology, Department of Medicine, Northwestern University Feinberg School of Medicine, Chicago, Illinois

**Keywords:** biomarkers, heart failure, predictive value, risk factors, sex differences, BMI, body mass index, CRP, C-reactive protein, cTn, cardiac troponin, CV, cardiovascular, HF, heart failure, HFpEF, heart failure with preserved ejection fraction, HFrEF, heart failure with reduced ejection fraction, MI, myocardial infarction, NP, natriuretic peptide, UACR, urinary albumin-to-creatinine ratio

## Abstract

**Background:**

Whether cardiovascular (CV) disease risk factors and biomarkers associate differentially with heart failure (HF) risk in men and women is unclear.

**Objectives:**

The purpose of this study was to evaluate sex-specific associations of CV risk factors and biomarkers with incident HF.

**Methods:**

The analysis was performed using data from 4 community-based cohorts with 12.5 years of follow-up. Participants (recruited between 1989 and 2002) were free of HF at baseline. Biomarker measurements included natriuretic peptides, cardiac troponins, plasminogen activator inhibitor-1, D-dimer, fibrinogen, C-reactive protein, sST2, galectin-3, cystatin-C, and urinary albumin-to-creatinine ratio.

**Results:**

Among 22,756 participants (mean age 60 ± 13 years, 53% women), HF occurred in 2,095 participants (47% women). Age, smoking, type 2 diabetes mellitus, hypertension, body mass index, atrial fibrillation, myocardial infarction, left ventricular hypertrophy, and left bundle branch block were strongly associated with HF in both sexes (p < 0.001), and the combined clinical model had good discrimination in men (C-statistic = 0.80) and in women (C-statistic = 0.83). The majority of biomarkers were strongly and similarly associated with HF in both sexes. The clinical model improved modestly after adding natriuretic peptides in men (ΔC-statistic = 0.006; likelihood ratio chi-square = 146; p < 0.001), and after adding cardiac troponins in women (ΔC-statistic = 0.003; likelihood ratio chi-square = 73; p < 0.001).

**Conclusions:**

CV risk factors are strongly and similarly associated with incident HF in both sexes, highlighting the similar importance of risk factor control in reducing HF risk in the community. There are subtle sex-related differences in the predictive value of individual biomarkers, but the overall improvement in HF risk estimation when included in a clinical HF risk prediction model is limited in both sexes.

Heart failure (HF) is a major public health problem and a leading cause of morbidity and mortality worldwide (1, 2, 3). Although lifetime risk estimates for HF are comparable in both sexes, at about 20% (4,5), the biological response to HF precursors is fundamentally different among women and men. For instance, after ischemic myocardial injury, adverse cardiac remodeling is more commonly observed in men than women (6,7). When subjected to pressure or volume overload, female hearts hypertrophy more than male hearts and tend to remodel in a concentric pattern, whereas male hearts more often display an eccentric remodeling pattern (8, 9, 10, 11, 12, 13). The exact mechanisms leading to the observed sex-related differences in HF pathogenesis are poorly understood.

Circulating biomarkers reflect distinct pathophysiological processes (14), and elevated levels of HF-related biomarkers may indicate cardiovascular (CV) or systemic derangement early in the time course of disease progression (15, 16, 17). For example, cardiac natriuretic peptide (NP) levels reflect myocardial stretch due to volume overload, whereas higher levels of cardiac troponins (cTns) indicate ongoing myocardial injury. Plasminogen activator inhibitor (PAI)-1, D-dimer, and fibrinogen levels represent thrombotic/fibrinolytic pathways; C-reactive protein (CRP) levels reflect systemic inflammation; galectin-3 and soluble interleukin-1 receptor-like 1 (sST2) levels indicate tissue fibrosis; and cystatin-C levels reflect renal function (15, 16, 17). Plasma concentrations of many of these biomarkers have been shown to differ between women and men: NPs, D-dimer, CRP, and galectin-3 are higher in women, whereas cTns and sST2 are higher in men (18, 19, 20, 21). Examining sex-specific associations of circulating biomarkers with incident HF may provide a deeper understanding of sex-specific mechanisms leading to HF and may facilitate the development of sex-specific risk prediction models.

To address the potential differences between men and women, we leveraged data from 4 well-characterized, community-based longitudinal cohorts with adjudicated HF endpoints: the FHS (Framingham Heart Study), the PREVEND (Prevention of REnal and Vascular End-stage Disease), the MESA (Multi-Ethnic Study of Atherosclerosis), and the CHS (Cardiovascular Health Study). Our objectives were to examine: 1) sex-specific associations of CV risk factors and biomarkers with incident HF; and 2) the extent to which individual biomarkers improve HF risk prediction in men and women.

## Methods

Individual-level data from 4 cohorts were harmonized and pooled, generating an initial total of 24,803 participants (22,23). The 4 cohorts included FHS offspring cohort examination 6 (1995 to 1998), PREVEND examination 1 (1997 to 1998), MESA examination 1 (2000 to 2002), and CHS examination 1 (1989 to 1990; 1992 to 1993 for a supplemental predominantly African-American cohort). From this sample, individuals were excluded for the following reasons: 1) prevalent HF (n = 326); 2) age <30 years (n = 124); 3) missing clinical covariates (n = 1,570); or 4) unavailable follow-up data (n = 27), resulting in a total of 22,756 individuals for the current analysis (Supplemental Figure 1). Written informed consent was obtained for all study participants. Appropriate institutional review board approval was obtained for all 4 cohorts (from Boston University [FHS]; University of Groningen [PREVEND]; Columbia University, Northwestern University, University of California–Los Angeles, and University of Minnesota [MESA]; Johns Hopkins University and Wake Forest University [MESA and CHS]; and University of California–Davis and University of Pittsburgh [CHS]).

### Clinical assessment, biomarker assays, and incident HF

Baseline examination included a detailed medical history, physical examination, fasting blood draw, and electrocardiography. Clinical risk factors were evaluated and harmonized across cohorts as previously described (22). Blood pressure (BP) was taken as the mean of 2 seated measurements. Hypertension was defined as systolic BP of 140 mm Hg or higher, diastolic BP of 90 mm Hg or higher, or antihypertensive medication usage. Body mass index (BMI) was calculated as weight/height^2^ (kg/m^2^). Diabetes mellitus was defined as a fasting glucose level ≥126 mg/dl (7.0 mmol/l), random glucose level ≥200 mg/dl (11.1 mmol/l), or hypoglycemic medication usage. Electrocardiography-assessed left ventricular hypertrophy (LVH) was defined based on accepted voltage and ST-segment criteria as previously described (22,23). Given that LVH and left bundle branch block (LBBB) were mutually exclusive electrocardiographic diagnoses, analyses were conducted using a 3-level categorical variable to represent LVH, LBBB or neither (22).

The current study included the following biomarkers: B-type natriuretic peptide (BNP)/N-terminal pro-BNP, high-sensitivity cTnT or cTnI, PAI-1, D-dimer, fibrinogen, high-sensitivity CRP, galectin-3, sST2, cystatin-C, and urinary albumin-to-creatinine ratio (UACR). These biomarkers were measured in at least 3 of the 4 cohorts, except sST2, which was measured in 2 cohorts. Details on biomarker availability across cohorts are shown in Supplemental Figure 2. BNP and cTnI were measured in FHS, whereas N-terminal pro-BNP and cTnT were measured in PREVEND, MESA, and CHS.

Individuals were followed prospectively for HF development or death. Adjudication of events was performed by study investigators within each cohort using established protocols after review of all available outpatient and hospital records. Incident HF was defined according to signs and symptoms as previously described (22,23). Medical records were reviewed for assessment of left ventricular (LV) function at or around the time of first HF event. HF events were subclassified into heart failure with preserved ejection fraction (HFpEF, LV ejection fraction ≥50%) or heart failure with reduced ejection fraction (HFrEF, LV ejection fraction <50%) based on echocardiography in >85% of HF cases, and as unclassified HF if LV function assessment was unavailable.

### Statistical analysis

All biomarker concentrations were natural log-transformed and standardized within each cohort to account for interassay and cohort-specific factors. Individual-level data from each of the 4 cohorts were then pooled for subsequent analyses (22,23). Follow-up time was truncated at 15 years. Continuous variables were presented as mean ± SD, and categorical variables were represented as counts (percentages). Baseline characteristics were compared among men and women using chi-square test for categorical variables. Age-adjusted linear regression models were employed to examine associations of individual biomarkers with sex.

In primary analyses, we evaluated sex-specific associations of clinical covariates and biomarkers with incident HF using Fine-Gray proportional subdistribution hazards models, accounting for death as a competing risk (24). First, we constructed sex-specific clinical models using the following variables based on previous publication (22): age, smoking, diabetes mellitus, hypertension, BMI, atrial fibrillation (additionally added), MI, and the presence of LVH/LBBB. We formally tested for *sex•covariate* interactions in sex-pooled models. A p value of 0.05 (multivariable model), and interaction p value of 0.01 (i.e., 0.1 divided by 9, Bonferroni adjustment) were used to designate statistical significance. Results with interaction p values between 0.01 and 0.1 were considered suggestive. Discrimination of the clinical HF model (Harrell’s C-statistic) was calculated separately in men and in women. We then examined sex-specific associations of individual biomarkers with incident HF after adjusting for clinical covariates. To facilitate clinical interpretation, we also performed a similar analysis using log_2_ transformed biomarkers, where results can be interpreted as HF risk per doubling of biomarker values. We formally tested for *biomarker•sex* interactions in sex-pooled models. A p value of 0.005 (i.e., 0.05 divided by 10) and interaction p value of 0.01 (i.e., 0.1 divided by 10) were used to designate statistical significance. Results with p values between 0.005 and 0.05 and interaction p values between 0.01 and 0.1 were considered suggestive.

In secondary analyses, biomarker models were also adjusted for NPs. For additional secondary analyses, those biomarkers displaying statistically significant associations with HF in the total population (independent of NPs) were selected along with NPs. We compared associations of selected biomarkers with HF subtypes (HFpEF vs. HFrEF) in men and in women using the Lunn-McNeil method (25), and Fine-Gray models accounted for the competing risk of death, other HF subtype, and unclassified HF (22,23). We used Harrell's C-statistic and likelihood ratio (LHR) test to examine the incremental predictive value of selected biomarkers (available in all 4 cohorts) over the clinical HF model in men and in women separately.

All models included a strata statement to account for study cohort, as well as for stratified recruitment in the PREVEND study (24-h urinary albumin excretion ≥10 mg/l vs. <10 mg/l) (22,23). All statistical analyses were conducted with SAS version 9.4 software (SAS Institute, Cary, North Carolina).

## Results

Of 22,756 participants (12,087 [53.1%] women), 989 women (8.1%) and 1,106 men (10.4%) developed HF over a median (Q1 to Q3) follow-up of 12.6 years (11.6 to 13.6 years) and 12.4 years (9.7 to 13.1 years), respectively. This resulted in an overall HF incidence of 7.1 (95% confidence interval [CI]: 6.6 to 7.5) per 1,000 person-years in women and 9.5 (95% CI: 8.9 to 10.1) per 1,000 person-years in men. Overall HF risk was also lower in women than men (multivariable-adjusted hazard ratio [HR]: 0.75; 95% CI: 0.68 to 0.82). Median (Q1 to Q3) time to HF diagnosis was 8.2 years (4.8 to 10.8 years) in women and 7.1 years (3.7 to 10.2 years) in men. Mean ages of women and men at the time of HF diagnosis were 79.6 ± 8.3 years and 77.3 ± 8.9 years, respectively.

### Sex-specific associations of CV risk factors

Sex differences in clinical characteristics are shown in Table 1 (cohort-specific characteristics in Supplemental Table 1). MI and atrial fibrillation were approximately twice as prevalent in men than women (p < 0.001). Hypertension, diabetes mellitus, and smoking history were also slightly more common in men (p < 0.005), whereas LVH was more commonly observed in women (p < 0.001).

**Table 1:** Baseline Characteristics in the Pooled Cohort: Men Versus Women

	Men (n = 10,669)	Women (n = 12,087)
Age, yrs	60 ± 13	60 ± 13
Race/ethnicity		
White	8,235 (77.4)	9,228 (76.5)
Black	1,155 (10.9)	1,526 (12.7)
Others	1,251 (11.8)	1,306 (10.8)
Medical history		
Smoking	2,215 (21)	2,326 (19)
Diabetes mellitus	1,193 (11)	1,073 (9)
Hypertension	4,950 (46)	5,320 (44)
Atrial fibrillation	178 (1.7)	86 (0.7)
Myocardial infarction	622 (6)	314 (3)
Clinical covariates		
Body mass index, kg/m^2^	27.1 ± 4.1	27.2 ± 5.6
Cholesterol, mg/dl	202 ± 41	212 ± 41
High-density lipoprotein cholesterol, mg/dl	45 ± 12	58 ± 16
Left ventricular hypertrophy	247 (2)	470 (4)
Left bundle branch block	82 (1)	89 (1)

Values are mean ± SD or n (%).

In both sexes, clinical risk factors included in the multivariable model were significantly associated with future HF risk (p < 0.001) (Table 2). When formally tested for interaction, only the *age•sex* term was significant (i.e., sex modified the effect of age significantly) (p_int_ = 0.001). Hypertension and BMI displayed suggestive interactions with sex (p_int_ = 0.07 and 0.02, respectively). Specifically, the risk of developing HF was more than 2-fold higher per 10 years of age among women (HR: 2.07; 95% CI: 1.89 to 2.28) compared with a 1.8-fold higher risk among men (HR: 1.80; 95% CI: 1.67 to 1.95). Similarly, the presence of hypertension portended a 98% higher risk of developing HF among women compared with a 67% higher risk among men. By contrast, a 4-kg/m^2^ increase in BMI was associated with a 28% higher risk of developing HF in men compared with an 18% higher risk among women. Discrimination of the clinical HF model, as ascertained by the C-statistic, was strong in men (C-statistic: 0.80; 95% CI: 0.79 to 0.82), and in women (C-statistic: 0.83; 95% CI: 0.82 to 0.84). Cohort-specific analyses are provided in Supplemental Table 2.

**Table 2:** Associations of Clinical Risk Factors With Incident Heart Failure: Men Versus Women

	Men		Women		Interaction
	sHR (95% CI)	p Value	sHR (95% CI)	p Value	p_int_
Age (per 10 yrs)	1.80 (1.67–1.95)	<0.001	2.07 (1.89–2.28)	<0.001	0.001
Smoking	1.36 (1.14–1.63)	0.001	1.50 (1.25–1.81)	<0.001	0.845
Diabetes mellitus	1.49 (1.28–1.72)	<0.001	1.76 (1.49–2.09)	<0.001	0.164
Hypertension	1.67 (1.45–1.93)	<0.001	1.98 (1.68–2.34)	<0.001	0.073
Body mass index (per 4 kg/m^2^)	1.28 (1.21–1.36)	<0.001	1.18 (1.12–1.24)	<0.001	0.020
Atrial fibrillation	1.83 (1.37–2.44)	<0.001	2.58 (1.62–4.13)	<0.001	0.153
Myocardial infarction	2.19 (1.85–2.60)	<0.001	1.69 (1.28–2.22)	<0.001	0.349
Left ventricular hypertrophy	2.11 (1.62–2.75)	<0.001	1.76 (1.36–2.26)	<0.001	0.515
Left bundle branch block	2.43 (1.62–3.63)	<0.001	3.14 (2.13–4.64)	<0.001	0.281
C-statistic	0.80 (0.79–0.82)	—	0.83 (0.81–0.84)	—	—

Fine-Gray models were adjusted for the competing risk of death, and for the following variables: age, smoking, diabetes mellitus, hypertension, body mass index, atrial fibrillation, myocardial infarction, and left ventricular hypertrophy/left bundle branch block; strata statement included. Interaction p value (p_int_) denotes *sex•covariate* interaction on a multiplicative scale in the total population.

CI = confidence interval; sHR = subdistribution hazard ratio per unit change in the clinical covariate.

### Sex-specific associations of CV biomarkers

Baseline NPs, D-dimer, fibrinogen, CRP, galectin-3, and UACR levels were higher in women (p < 0.001). By contrast, cTns, PAI-1, sST2, and cystatin-C levels were higher in men (p < 0.001). These sex-related differences were largely consistent across cohorts (Supplemental Table 3, Supplemental Figure 3). In Fine-Gray survival models, all biomarkers except PAI-1 and sST2 were significantly associated with incident HF in women (p value for each ≤0.005), whereas all biomarkers except PAI-1, sST2, and galectin-3 were significantly associated with incident HF in men (p value for each <0.001). None of the biomarkers showed a significant interaction with sex for incident HF (Central Illustration, Supplemental Table 4). Galectin-3 displayed a suggestive interaction with sex (p_int_ = 0.04), and was significantly associated with incident HF only in women (HR: 1.13; 95% CI: 1.05 to 1.22). Our results did not materially change when we used log_2_ transformed biomarker values (Supplemental Table 5). Cohort-specific analyses are provided in Supplemental Table 6.

**Central Illustration:**
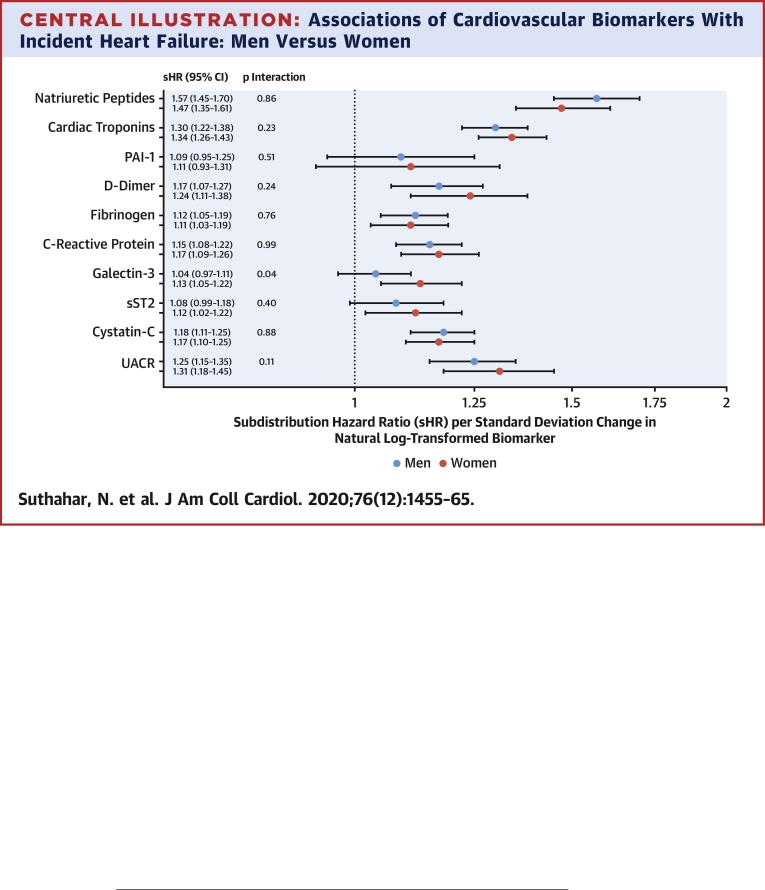
Associations of Cardiovascular Biomarkers With Incident Heart Failure: Men Versus Women Associations of individual biomarkers with incident heart failure were evaluated using Fine-Gray models adjusting for the competing risk of death, and for the following variables: age, smoking, diabetes mellitus, hypertension, body mass index, atrial fibrillation, myocardial infarction, and presence of left ventricular hypertrophy/left bundle branch block. Natriuretic peptides include N-terminal pro–B-type natriuretic peptide or B-type natriuretic peptide. Cardiac troponins include cardiac troponin-T or I. Interaction p value denotes *sex•covariate* interaction on a multiplicative scale in the total population. None of the biomarkers displayed a significant interaction with sex for heart failure outcome. CI = confidence interval; PAI = plasminogen activator inhibitor; sHR = subdistribution hazard ratio; sST2 = soluble interleukin-1 receptor-like 1; UACR = urinary albumin-to-creatinine ratio.

NPs were strongly associated with incident HF in both men (HR: 1.57; 95% CI: 1.45 to 1.70) and women (HR: 1.47; 95% CI: 1.35 to 1.61) with no substantial sex-related differences (p_int_ = 0.86). The *NP•BMI* interaction term was not significant in both sexes. The *NP•age* interaction term was significant in both men (HR_int_: 0.88; 95% CI: 0.82 to 0.94; p_int_ < 0.001) and women (HR_int_: 0.88; 95% CI: 0.81 to 0.94; p_int_ < 0.001). After further adjustment for NPs, 3 biomarkers remained significantly associated with incident HF in the total population: cTns, CRP, and UACR (p ≤ 0.001). We examined the shape of these associations using restricted cubic spline models (Supplemental Figure 4). In sex-specific analyses, only cTns remained significantly associated with HF in men (HR: 1.15; 95% CI: 1.07 to 1.23), whereas all 3 biomarkers remained associated with HF in women (HR_cTns_: 1.25; 95% CI: 1.17 to 1.34; HR_CRP_: 1.14; 95% CI: 1.05 to 1.24; HR_UACR_: 1.27; 95% CI: 1.14 to 1.41) (Table 3). Associations of selected biomarkers with HF subtypes (HFpEF vs. HFrEF) in men and in women are shown in Table 4. Finally, we evaluated the sex-specific incremental predictive value of selected biomarkers (available in all 4 cohorts) over the clinical HF model (Table 5). The addition of individual biomarkers (i.e., NPs, cTns, and CRP) did not appreciably improve model discrimination in both sexes, with the greatest increment observed after adding NPs in men (ΔC-statistic: 0.006), and after adding cTns and CRP in women (ΔC-statistic: 0.003). NPs and cTns improved model fit modestly in men (LHR chi-square for NPs: 146; p < 0.001, and LHR chi-square for cTns: 67; p < 0.001), and in women (LHR chi-square for NPs: 83; p < 0.001, and LHR chi-square for cTns: 73; p < 0.001, respectively).

**Table 3:** Associations of Individual Biomarkers With Incident Heart Failure After Adjusting for Natriuretic Peptides

	Total		Men		Women	
	sHR (95% CI)	p Value	sHR (95% CI)	p Value	sHR (95% CI)	p Value
Cardiac troponins	1.20 (1.14–1.26)	<0.001	1.15 (1.07–1.23)	<0.001	1.25 (1.17–1.34)	<0.001
D-dimer	1.11 (1.02–1.20)	0.01	1.08 (0.98–1.20)	0.129	1.17 (1.03–1.33)	0.014
Fibrinogen	1.07 (1.02–1.13)	0.01	1.06 (0.99–1.14)	0.112	1.10 (1.01–1.19)	0.023
C-reactive protein	1.09 (1.03–1.15)	0.001	1.07 (1.00–1.15)	0.06	1.14 (1.05–1.24)	0.001
sST2	1.04 (0.97–1.12)	0.243	1.01 (0.91–1.11)	0.857	1.07 (0.97–1.18)	0.157
Galectin-3	1.01 (0.95–1.06)	0.808	0.97 (0.90–1.04)	0.368	1.07 (0.99–1.16)	0.107
Cystatin-C	1.04 (0.99–1.09)	0.136	1.01 (0.95–1.08)	0.793	1.06 (0.99–1.14)	0.086
UACR	1.15 (1.07–1.23)	<0.001	1.10 (1.01–1.20)	0.027	1.27 (1.14–1.41)	<0.001

Fine-Gray models were adjusted for the competing risk of death, and for the following variables: age, smoking, diabetes mellitus, hypertension, body mass index, atrial fibrillation, myocardial infarction, presence of left ventricular hypertrophy/left bundle branch block, and natriuretic peptides; strata statement included. Models evaluating associations with incident heart failure in the combined (male + female) population were also adjusted for sex.

CI = confidence interval; sHR = subdistribution hazard ratio per standard deviation change in natural log-transformed biomarker; sST2 = soluble interleukin-1 receptor like 1; UACR = urinary albumin-to-creatinine ratio.

**Table 4:** Sex-Specific Associations of Selected Biomarkers With Heart Failure Subtypes (HFpEF vs. HFrEF)

	Men		p_equality_	Women		p_equality_
	HFpEF	HFrEF		HFpEF	HFrEF	
	sHR (95% CI)	sHR (95% CI)		sHR (95% CI)	sHR (95% CI)	
Natriuretic peptides	1.32 (1.15–1.50)	1.64 (1.50–1.81)	0.012	1.30 (1.15–1.47)	1.46 (1.24–1.72)	0.289
Cardiac troponins	1.03 (0.92–1.15)	1.40 (1.30–1.50)	<0.001	1.18 (1.07–1.30)	1.36 (1.22–1.51)	0.048
C–reactive protein	1.02 (0.90–1.17)	1.16 (1.06–1.27)	0.117	1.07 (0.95–1.21)	1.33 (1.18–1.50)	0.018
UACR	1.30 (1.14–1.49)	1.21 (1.10–1.34)	0.452	1.35 (1.15–1.58)	1.16 (0.99–1.35)	0.181

Fine-Gray models adjusted for competing risk of death, other HF subtype, and unclassified HF, and for the following variables: age, smoking, diabetes mellitus, hypertension, body mass index, atrial fibrillation, myocardial infarction, and left ventricular hypertrophy/left bundle branch block; strata statement included.

HFpEF = heart failure with preserved ejection fraction; HFrEF = heart failure with reduced ejection fraction; other abbreviations as in Table 3.

**Table 5:** Sex-Specific Incremental Value of Selected Biomarkers Over the Clinical Model

Biomarkers	Men	p Value	Women	p Value
	Risk Estimation		Risk Estimation	
C-statistic ∗	0.797 (0.784–0.809)	—	0.815 (0.804–0.827)	—
LHR∗	12376	—	11991	—
Natriuretic peptides				
C-statistic + NPs	0.803 (0.790–0.815)	—	0.811 (0.799–0.823)	—
Δ C-statistic	0.006	—	−0.004	—
LHR + NPs	12,230	—	11,908	—
LHR chi-square	146	<0.001	83	<0.001
Cardiac troponins				
C-statistic + cTns	0.800 (0.787–0.813)	—	0.818 (0.806–0.829)	—
Δ C-statistic	0.003	—	0.003	—
LHR + hs-Tn	12,309	—	11,918	—
LHR chi-square	67	<0.001	73	<0.001
C-reactive protein				
C-statistic + CRP	0.798 (0.785–0.810)	—	0.818 (0.806–0.829)	—
Δ C-statistic	0.001	—	0.003	—
LHR + CRP	12,367	—	11,976	—
LHR chi-square	9	0.003	15	<0.001

For these analyses, 8,926 men with 879 HF events and 9,328 women with 830 HF events with no missing biomarker measurements were included.

CRP = C-reactive protein; cTn = cardiac troponin; LHR = likelihood ratio test; NP = natriuretic peptide.

∗ Base model includes age, smoking, diabetes mellitus, hypertension, body mass index, atrial fibrillation, myocardial infarction, and presence of left ventricular hypertrophy/left bundle branch block; strata statement included.

## Discussion

In the current study, we examined sex-specific associations of CV risk factors and biomarkers with incident HF in 22,756 individuals from 4 longitudinal community-based cohorts. Our principal findings are as follows: 1) CV risk factors were strongly associated with incident HF in both sexes with minor sex-related differences; 2) the majority of biomarkers were strongly and similarly associated with incident HF in both sexes (Central Illustration); and 3) subtle sex-related differences were observed in the prognostic value of individual biomarkers, but the overall improvement in HF risk prediction was limited in men and in women.

### Sex-specific associations of CV risk factors

Clinical risk factors were strongly associated with HF risk in both sexes, and only minor sex-related differences were observed in our study. Specifically, higher age was more strongly associated with HF risk in women. Stronger associations of age with HF risk in women could potentially reflect sex-related differences in HF incidence in the elderly: crude HF incidence is higher in women than men in older age groups (>80 years) (3). In this context, it is essential to consider that death precludes individuals from developing HF (26), and men (on an average) die at a younger age than women (3,27, 28, 29). However, our models adjusted for the competing risk of death. This suggests that other CV risk factors not included in our study (e.g., microvascular disease [30]) may be more strongly associated with higher age in women than men. Furthermore, it is known that women have higher systolic and diastolic LV elastance than men at a given age, and the differences (particularly for end-diastolic elastance) are accentuated with aging (31,32), which could potentially explain stronger associations of age with incident HF in women.

Hypertension also tended to be more strongly associated with HF risk in women than men in our study. These data should, however, be interpreted along with the findings from previous studies. For instance, a U.S.-based study also found that higher systolic BP related more strongly with HF risk in women (Black and White) than men (33). By contrast, a European study including approximately 80,000 individuals showed that systolic BP was more strongly associated with incident HF in men than women; the population attributable risk of hypertension was also higher in men (34). Interestingly, a U.K.-based study showed that associations of hypertension with incident HF was modest in both sexes, although the relative contribution of hypertension to HF risk was again higher in men (35). Taken together, these results indicate that sex-related differences in associations of hypertension with incident HF vary considerably depending on cohort selection.

Next, we observed that BMI tended to be more strongly associated with HF risk in men than women. Stronger associations of BMI with incident HF in men should also be interpreted cautiously and viewed in the context of 4 large recently published studies (33, 34, 35, 36). In the study conducted by Khan et al. (33) using pooled data from 5 large U.S.-based cohorts, BMI was a stronger determinant of HF risk in White men compared with White women, and in Black women compared with Black men. However, in a more recent study on high-risk, low-income individuals from southeastern United States, being overweight (BMI ≥25 kg/m^2^) was significantly associated with incident HF only in White men and women, but not in Black individuals (36). In a U.K.-based study using electronic health records data from over 800,000 individuals, the relative contribution of obesity to HF risk appeared to be higher in women than men, particularly in younger individuals (age 55 to 65 years) (35). Likewise, in a study examining pooled data from several European community-based cohorts, the population attributable risk of obesity (BMI ≥30 kg/m^2^) was higher in women than men, although BMI was strongly and similarly associated with HF risk in both sexes (34).

Finally, in the current study, prevalent MI was similarly associated with HF risk in both sexes. Nevertheless, the population attributable fraction of MI to incident HF would still be higher in men than women, due to the substantially higher prevalence of MI in men. In a recent European population-based study, prevalence of MI, as well as the population attributable fraction of MI to incident HF, were higher in men than in women (34). Similar trends were also reported in a U.S.-based study in both Black and White subpopulations (36). Although identifying myocardial injury based on a universal cutpoint versus sex-specific cutpoints (37) could have affected prevalence rates of MI to some extent, these data collectively suggest that the overall contribution of MI to the population burden of HF is higher in men than women.

### Sex-specific associations of CV biomarkers

One of the reasons for examining sex-specific associations of circulating biomarkers with incident HF in community-dwelling individuals was to improve our understanding of sex-specific mechanisms related to HF risk. Contrary to expectation, our study demonstrated that major HF-related pathophysiological mechanisms sensed by biomarkers were, in fact, broadly similar in women and men (Central Illustration). Nevertheless, there are 2 points worth discussing. First, biomarker levels differed substantially between men and women (Supplemental Figure 3), and similar results were consistently observed across multiple community-based cohorts (18, 19, 20, 21). For most biomarkers, baseline sex-related differences need not indicate sex-specific pathophysiology but may rather be a manifestation of physiological sex-based differences (21). Second, despite strikingly similar associations of most biomarkers with incident HF in both sexes, we did observe sex-related differences in associations of profibrotic marker galectin-3 with incident HF (i.e., an equivalent increment in galectin-3 levels within the population tended to be more strongly associated with HF risk in women than men). Future studies are needed to understand whether fibrotic mechanisms (e.g., vascular, pulmonary, skeletal muscle, and cardiac fibrosis) may play a greater role in the pathophysiology of HF in women.

Notably, in our analysis, NPs were strongly and similarly associated with incident HF in both sexes. However, previous studies (38) including the study conducted by Magnussen et al. (34) indicate that higher NP levels related more strongly with HF risk in men than women. A potential explanation for the discrepancy in results could be that the current study used Fine-Gray subdistribution hazards models accounting for death as a competing risk, and also adjusted for cardiac risk factors such as atrial fibrillation, LVH, and LBBB. Assay-related effects and cohort heterogeneity could be other factors influencing our results. However, differences in effect sizes (men vs. women) within individual cohorts as well as between cohorts were modest with the greatest variability observed between CHS and the remaining cohorts. Interestingly, in the pooled cohort, we observed that with increasing age, associations of NPs with incident HF significantly declined, but this age-related effect was comparable in both sexes.

When biomarker models were further adjusted for NPs, only cTns remained significantly associated with incident HF in men. On the other hand, cTns, hs-CRP, and UACR remained significantly associated with incident HF in women. These findings highlight the independent predictive value of cTns beyond NPs in both sexes. From a biological perspective, our results suggest that systemic inflammation and renal dysfunction may play a greater role in the pathophysiology of HF in women. However, given that most of the biomarkers were similarly associated with HF risk in both sexes in our primary analyses, these data should be viewed only as hypothesis-generating.

### Sex-specific associations of selected biomarkers with HF subtypes

Previously, we reported that the majority of CV biomarkers (except UACR) were more strongly associated with HFrEF than HFpEF (23). We now show that these findings are generally valid for both sexes. Specifically, cTns and NPs were more strongly associated with HFrEF in men, and cTns and CRP were more strongly associated with HFrEF in women. UACR was similarly associated with HF subtypes, but displayed robust associations with HFpEF in both sexes. Nevertheless, due to limited statistical power to detect differences in the subanalysis, and given that UACR measurements were available only in 3 cohorts, these results should be cautiously interpreted.

### Sex-specific predictive value of selected biomarkers

Only subtle sex-related differences were observed in the predictive value of individual biomarkers for incident HF. For instance, the greatest improvement in model fit was observed after adding NPs in men, whereas both NPs and cTns improved model fit to a similar extent in women. The addition of individual biomarkers, however, did not result in clinically relevant increments to model discrimination in both sexes, with NPs even slightly reducing model discrimination in women. Collectively, these data indicate that the value of individual biomarkers to improve HF risk prediction is limited in both sexes, highlighting the fact that the current clinical model is robust and sufficient to predict incident HF in both men and women.

### Study limitations

First, although HF endpoints were adjudicated in all 4 cohorts, a universal definition for HF is lacking (26), which may have influenced associations of clinical covariates as well as biomarkers with incident HF. Second, not all biomarkers were available in all cohorts. However, biomarkers selected for HF risk estimation were available in all 4 cohorts. Third, a single measurement of a biomarker may not effectively capture pathophysiology, given the large degree of interindividual and intraindividual variation (39). Although serial biomarker measurements could have provided us with more precise information, such data are not (yet) routinely available in large epidemiological studies. Fourth, we acknowledge that the C-statistic may not be an optimal tool to detect the incremental prognostic performance of a covariate over a well-established model, particularly when the base model is strong (40). These results should therefore be interpreted along with results from likelihood-ratio tests. Fifth, we chose 50% as the LVEF cutpoint that delineated HFrEF from HFpEF. This may be debated, but previous sensitivity analyses demonstrated only minor differences using an LVEF cutpoint of 45% (22). Further, prior studies also indicate that HF with LVEF between 40% and 50% resemble HFrEF more than HFpEF (41,42). Sixth, although MESA included individuals from multiple ethnicities, and CHS enrolled a supplemental, predominantly African-American cohort, the majority of participants in the pooled cohort were of European ancestry. This limits the generalizability of our findings to other races/ethnicities to some extent. Finally, our study was observational; therefore, residual confounding cannot be excluded, and we cannot establish causal relations among individual clinical risk factors, biomarkers, and HF.

## Conclusions

Our findings from 4 well-characterized community-based cohorts indicate that clinical risk factors are strongly and similarly associated with HF risk in both sexes. We also show that the majority of biomarkers remain strongly and similarly associated with incident HF in both sexes. However, the value of individual biomarkers, measured at a single time point, to improve HF risk prediction above and beyond an established clinical HF model is limited in both men and women.

Perspectives **COMPETENCY IN SYSTEMS-BASED PRACTICE:** The prevalence of CV risk factors and risk of developing HF are generally lower in women than in men. Once an individual risk factor or manifestation of heart disease (such as obesity, diabetes, hypertension, or MI) develops, however, the increase in HF risk is comparable across sexes. Likewise, although baseline biomarker levels differ between men and women, equal increases (e.g., 2-fold change) in biomarker levels are associated with similar increases in the risk of HF in both sexes.

**TRANSLATIONAL OUTLOOK:** Future prospective studies should examine sex-specific mechanisms related to HF pathogenesis.
