# Supplementary material for: Sex-Specific Associations of Cardiovascular Risk Factors and Biomarkers With Incident Heart Failure
Source: J Am Coll Cardiol. 2020 Sep 22;76(12):1455–65. doi: 10.1016/j.jacc.2020.07.044 (PMC7493711; doi:10.1016/j.jacc.2020.07.044)
Supplement: Supplemental Data [file mmc1.docx]

**Supplemental Figure 1. Participant Selection**

Abbreviations: FHS, Framingham Heart Study; PREVEND, Prevention of Renal and Vascular End-stage Disease; MESA, Multi-Ethnic Study of Atherosclerosis; CHS, Cardiovascular Health Study.

**Supplemental Figure 2. Biomarker availability across cohorts**
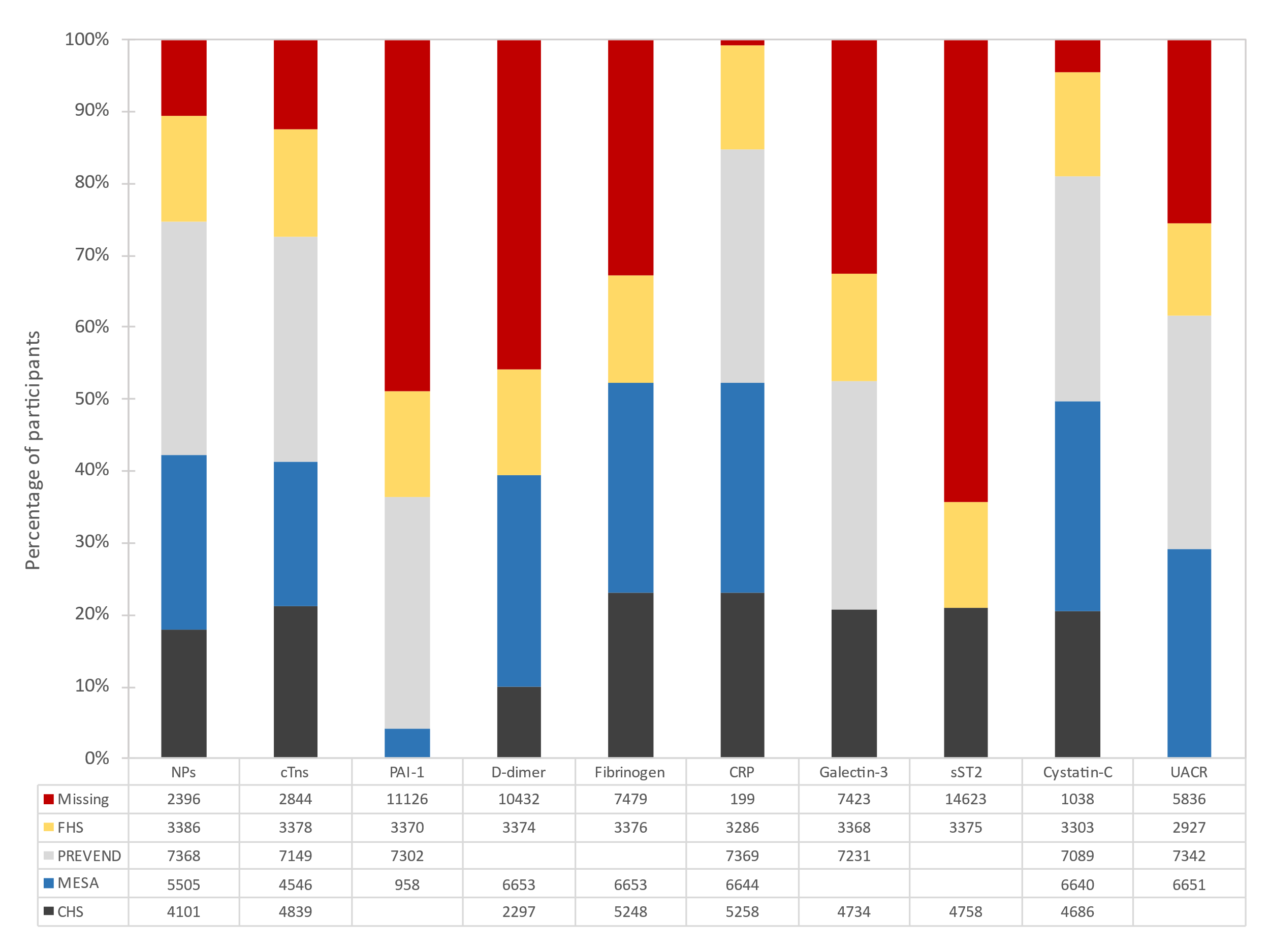


Abbreviations: FHS, Framingham Heart Study; PREVEND, Prevention of Renal and Vascular End-stage Disease; MESA, Multi-Ethnic Study of Atherosclerosis; CHS, Cardiovascular Health Study. NPs, natriuretic peptides; cTns, high-sensitivity cardiac troponins; PAI-1, plasminogen activator inhibitor-1; CRP, high-sensitivity C-reactive protein; sST2, interleukin-1 receptor-like 1; UACR, urinary albumin-to-creatinine ratio. For details on biomarker assays, we refer readers to reference 23 in the main manuscript.

**Supplemental Figure 3. Associations of cardiovascular biomarkers with sex**


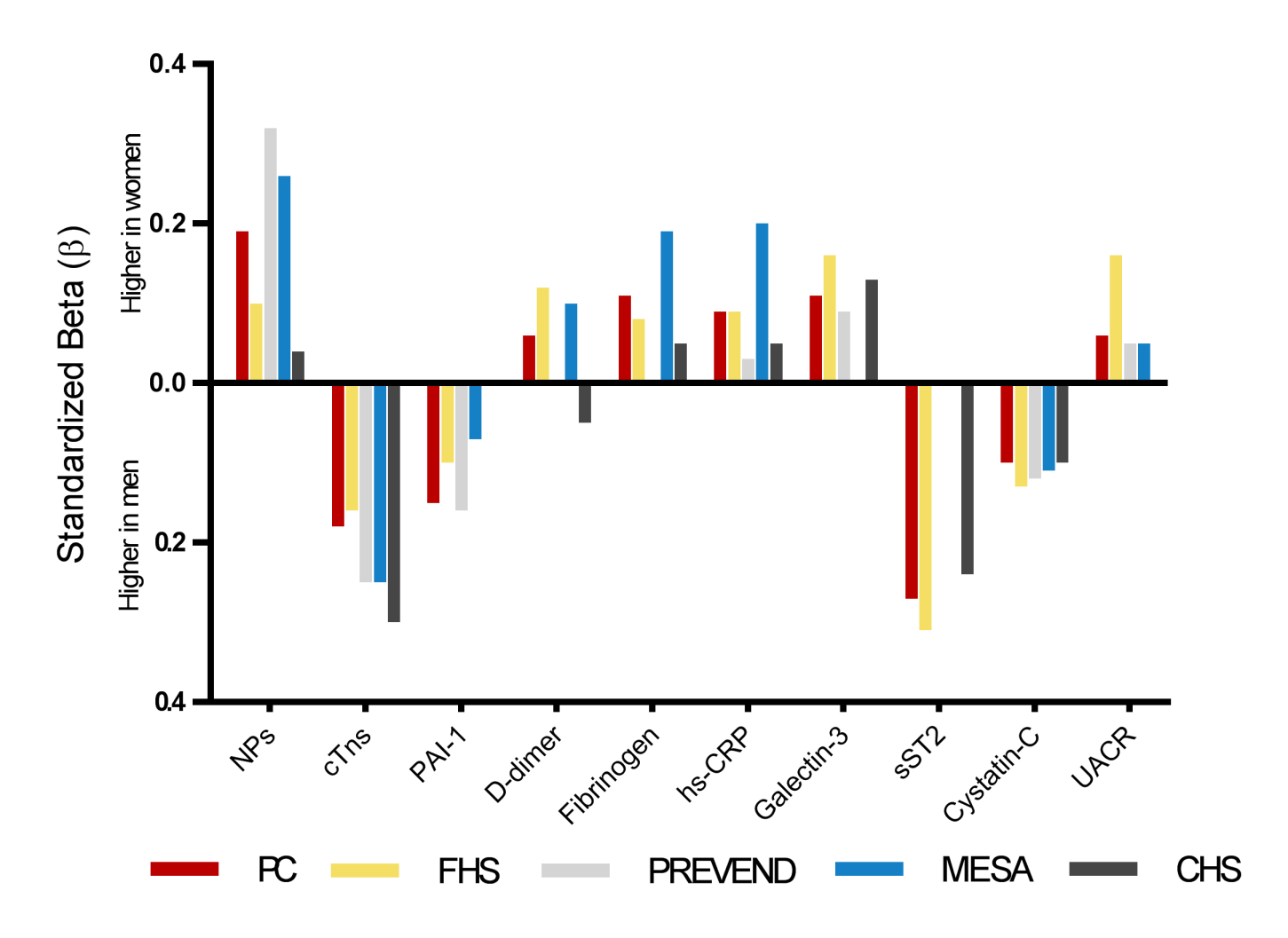


Individual biomarkers are depicted in the X-axis. Standardized beta (Sβ) coefficients, indicating associations of biomarker levels with sex, are shown in Y-axis. All models are adjusted for age. Domains represented by individual biomarkers include myocardial stretch (NPs), myocardial injury (cTns), thrombosis / fibrinolysis (PAI-1, D-dimer, fibrinogen), systemic inflammation (hs-CRP), tissue fibrosis (galectin-3, sST2) and renal function (cystatin-C, UACR). Abbreviations: PC, pooled cohort; FHS, Framingham Heart Study; PREVEND, Prevention of Renal and Vascular End-stage Disease; MESA, Multi-Ethnic Study of Atherosclerosis; CHS, Cardiovascular Health Study. NPs, natriuretic peptides; cTns, cardiac troponins; PAI-1, plasminogen activator inhibitor-1; hs-CRP, high-sensitivity C-reactive protein; sST2, interleukin-1 receptor-like 1; UACR, urinary albumin-to-creatinine ratio.

**Supplemental Figure 4. Associations of selected biomarkers with incident heart failure after adjusting for natriuretic peptides**

**
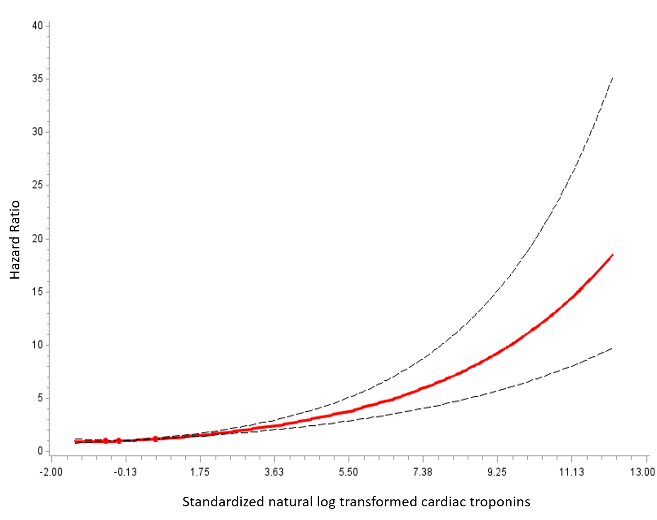

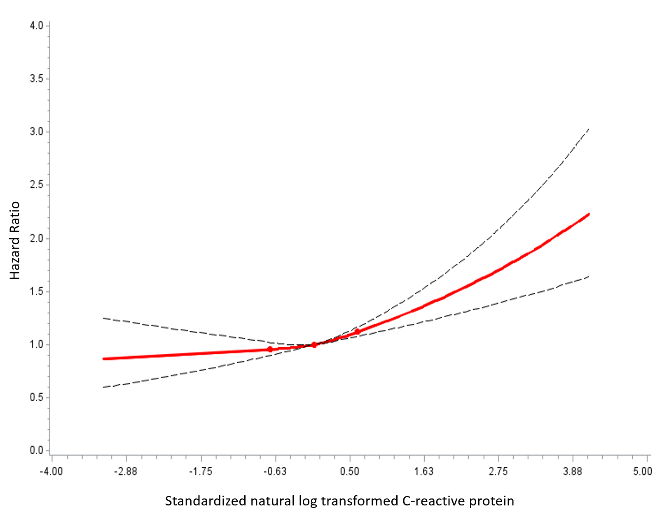

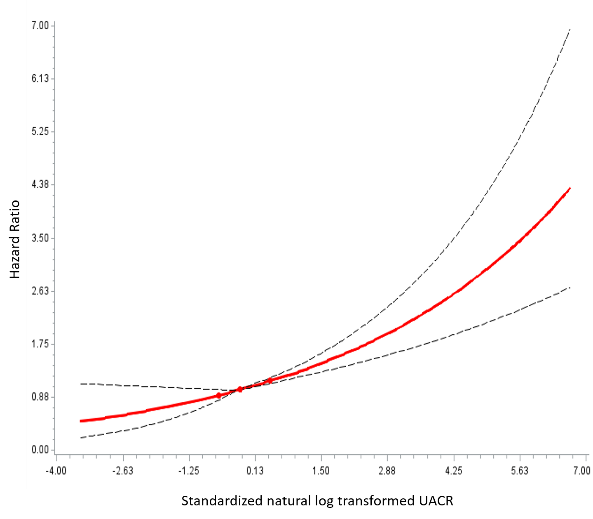
**

Spline regression models were knotted at 25^th^, 50^th^ and 75^th^ percentiles. Fine-Gray models accounted for the competing risk of death, and for the following variables: age, sex, smoking, diabetes mellitus, hypertension, body-mass index, myocardial infarction, atrial fibrillation, left ventricular hypertrophy / left bundle branch block and natriuretic peptides. Models did not significantly deviate from a linear trend. Abbreviation: UACR, urinary albumin-to-creatinine ratio.

| Biomarker | Minimum | Maximum | P(25) | P(50) | P(75) |
| --- | --- | --- | --- | --- | --- |
| Cardac troponins | -1.42 | 12.14 | -0.64 | -0.32 | 0.61 |
| C-reactive protein | -3.22 | 4.11 | -0.71 | -0.04 | 0.61 |
| UACR | -3.51 | 6.67 | -0.64 | 0.02 | 0.43 |

**Supplemental Table 1. Cohort-specific baseline characteristics in men and in women**

|  | **FHS** | | **PREVEND** | | **MESA** | | **CHS** | |
| --- | --- | --- | --- | --- | --- | --- | --- | --- |
| **Characteristics** | Men  (N= 1605) | Women  (N=1826) | Men  (N= 3667) | Women  (N=3702) | Men  (N= 3158) | Women  (N=3521) | Men  (N= 2239) | Women  (N=3038) |
| Age, years | 59 (10) | 59 (10) | 50 (13) | 48 (12) | 62 (10) | 62 (10) | 73 96) | 72 (5) |
| Race / ethnicity  White, N (%)  Black, N (%)  Others, N (%) | 1605 (100)  n/a  n/a | 1826 (100)  n/a  n/a | 3472 (95.4)  32 (0.9)  135 (3.7) | 3529 (96.0)  33 (0.9)  113 (3.1) | 1229 (38.9)  826 (26.2)  1103 (34.9) | 1332 (37.8)  1012 (28.7)  1177 (33.4) | 1929 (86.2)  297 (13.3)  13 (0.6) | 2541 (83.6)  481 (15.8)  16 (0.5) |
| Smoking, N (%) | 237 (15) | 288 (16) | 1271 (35) | 1247 (34) | 460 (15) | 412 (12) | 247 (11) | 379 (13) |
| Diabetes Mellitus, N (%) | 187 (12) | 147 (8) | 152 (4) | 120 (3) | 443 (14) | 398 (11) | 411 (18) | 408 (13) |
| Hypertension, N (%) | 701 (44) | 678 (37) | 1368 (37) | 969 (26) | 1482 (47) | 1719 (49) | 1399 (63) | 1954 (64) |
| Atrial fibrillation, N (%) | 70 (4.4) | 21 (1.2) | 46 (1.3) | 16 (0.4) | n/a | n/a | 61 (2.7) | 49 (1.6) |
| Myocardial infarction, N (%) | 93 (6) | 20 (1) | 268 (7) | 137 (4) | n/a | n/a | 261 (12) | 157 (5) |
| Body-mass index, kg/m^2^ | 28.5 (4.4) | 27.4 (5.7) | 26.3 (3.7) | 25.9 (4.7) | 27.9 (4.4) | 28.7 (6.2) | 26.4 (3.7) | 26.8 (5.2) |
| Cholesterol, mg/dL | 199 (41) | 212 (38) | 219 (43) | 217 (44) | 188 (35) | 200 (36) | 199 (36) | 221 (39) |
| HDL cholesterol, mg/dL | 43 (12) | 58 (16) | 45 (12) | 57 (16) | 45 (12) | 56 (15) | 48 (13) | 59 (16) |
| Left ventricular hypertrophy, N (%) | 57 (4) | 17 (1) | 44 (1) | 130 (4) | 48 (2) | 194 (6) | 98 (4) | 129 (4) |
| Left bundle branch block, N (%) | 15 (0.9) | 20 (1.1) | 18 (0.5) | 12 (0.3) | 14 (0.4) | 9 (0.3) | 35 (1.6) | 48 (1.6) |

Continuous data are presented as mean, standard deviation. Categorical data are presented as count (N), percentage (%)

**Supplemental Table 2. Cohort-specific associations of clinical risk factors with incident heart failure in men and in women**

|  | Subdistribution Hazard Ratio (95% Confidence Interval) | | | |
| --- | --- | --- | --- | --- |
|  | **FHS** | **PREVEND** | **MESA** | **CHS** |
| MEN |  |  |  |  |
| Age (per 10 years) | 2.31 (1.82-2.93) | 2.12 (1.66-2.72) | 1.80 (1.52-2.15) | 1.47 (1.28-1.69) |
| Smoking | 1.29 (0.75-2.23) | 1.50 (0.92-2.42) | 1.69 (1.10-2.59) | 1.25 (0.96-1.64) |
| Diabetes Mellitus | 1.73 (1.13-2.64) | 1.69 (0.82-3.52) | 1.77 (1.23-2.54) | 1.33 (1.10-1.62) |
| Hypertension | 1.24 (0.82-1.86) | 1.37 (0.77-2.42) | 2.20 (1.48-3.28) | 1.54 (1.28-1.85) |
| Body-mass index (per 4 kg/m^2^) | 1.34 (1.15-1.56) | 1.41 (1.14-1.74) | 1.26 (1.09-1.47) | 1.22 (1.13-1.33) |
| Atrial fibrillation | 2.08 (1.20-3.62) | 1.52 (0.78-2.96) | n/a | 1.60 (1.06-2.42) |
| Myocardial infarction | 2.57 (1.60-4.12) | 2.89 (1.73-4.83) | n/a | 1.99 (1.60-2.46) |
| Left ventricular hypertrophy | 4.55 (2.63-7.88) | 1.04 (0.35-3.03) | 2.90 (1.43-5.89) | 1.80 (1.28-2.53) |
| Left bundle branch block | 1.62 (0.49-5.32) | 2.02 (0.67-6.07) | 5.04 (1.75-14.48) | 2.34 (1.42-3.88) |
| WOMEN |  |  |  |  |
| Age (per 10 years) | 3.04 (2.18-4.23) | 2.96 (2.18-4.01) | 2.03 (1.62-2.57) | 1.72 (1.50-1.98) |
| Smoking | 1.59 (0.85-2.98) | 1.68 (0.93-3.05) | 1.75 (0.98-3.14) | 1.30 (1.02-1.65) |
| Diabetes Mellitus | 2.49 (1.47-4.22) | 1.57 (0.72-3.39) | 2.62 (1.71-4.02) | 1.55 (1.27-1.90) |
| Hypertension | 2.54 (1.49-4.32) | 1.86 (0.94-3.65) | 1.56 (0.96-2.53) | 1.79 (1.48-2.18) |
| Body-mass index (per 4 kg/m^2^) | 1.31 (1.12-1.53) | 1.31 (1.02-1.68) | 1.24 (1.09-1.41) | 1.14 (1.07-1.21) |
| Atrial fibrillation | 3.71 (1.38-9.97) | 3.90 (1.55-9.82) | n/a | 2.33 (1.38-3.94) |
| Myocardial infarction | 1.18 (0.37-3.83) | 1.84 (0.93-3.64) | n/a | 1.63 (1.20-2.20) |
| Left ventricular hypertrophy | 4.03 (1.29-12.59) | 1.63 (0.65-4.07) | 1.61 (0.86-3.00) | 1.78 (1.31-2.42) |
| Left bundle branch block | 2.38 (0.55-10.2) | 3.39 (0.66-17.41) | 9.86 (2.71-35.87) | 3.01 (1.96-4.62) |

Multivariable Fine-Gray models were adjusted for the competing risk of death, and included all of the following variables: age, smoking, diabetes mellitus, hypertension, body-mass index, atrial fibrillation, myocardial infarction and left ventricular hypertrophy / left bundle branch block. PREVEND models were adjusted for study design.

**Supplemental Table 3. Biomarker distribution in men and in women**

|  | **PC** | **FHS** | **PREVEND** | **MESA** | **CHS** |
| --- | --- | --- | --- | --- | --- |
| MEN |  |  |  |  |  |
| Natriuretic peptides | 3.42 (1.39) | 2.19 (0.92) | 3.23 (1.23) | 3.6 (1.22) | 4.68 (1.23) |
| Cardiac troponins | 1.58 (0.84) | 0.59 (0.73) | 1.42 (0.61) | 2.01 (0.58) | 2.09 (0.77) |
| PAI-1 | 3.99 (0.97) | 3.2 (0.58) | 4.45 (0.78) | 2.97 (0.88) | n/a |
| D-dimer | 5.38 (0.89) | 5.64 (0.7) | n/a | 5.31 (0.96) | 5.15 (0.86) |
| Fibrinogen | 5.77 (0.2) | 5.79 (0.2) | n/a | 5.79 (0.2) | 5.75 (0.2) |
| C-reactive protein | 0.53 (1.22) | 0.64 (1.1) | 0.4 (1.42) | 0.4 (1.09) | 0.87 (1.03) |
| Galectin-3 | 2.51 (0.34) | 2.58 (0.27) | 2.37 (0.3) | n/a | 2.69 (0.35) |
| sST2 | 3.24 (0.35) | 3.16 (0.34) | n/a | n/a | 3.3 (0.35) |
| Cystatin-C | -0.11 (0.26) | -0.07 (0.2) | -0.22 (0.29) | -0.11 (0.21) | 0 .04 (0.23) |
| UACR | 1.95 (1.29) | 1.48 (1.81) | 2.19 (1.05) | 1.89 (1.22) | n/a |
| WOMEN |  |  |  |  |  |
| Natriuretic peptides | 3.91 (1.25) | 2.38 (0.88) | 3.89 (0.95) | 4.23 (1.05) | 4.73 (1.09) |
| Cardiac troponins | 1.24 (0.74) | 0.35 (0.72) | 1.1 (0.42) | 1.76 (0.51) | 1.61 (0.65) |
| PAI-1 | 3.68 (0.99) | 2.99 (0.68) | 4.15 (0.84) | 2.85 (0.93) | n/a |
| D-dimer | 5.48 (0.83) | 5.79 (0.59) | n/a | 5.5 (0.89) | 5.03 (0.76) |
| Fibrinogen | 5.82 (0.21) | 5.82 (0.19) | n/a | 5.86 (0.21) | 5.76 (0.2) |
| C-reactive protein | 0.76 (1.24) | 0.86 (1.21) | 0.42 (1.38) | 0.87 (1.18) | 0.98 (1.03) |
| Galectin-3 | 2.59 (0.34) | 2.66 (0.26) | 2.41 (0.31) | n/a | 2.77 (0.31) |
| sST2 | 3.05 (0.35) | 2.94 (0.33) | n/a | n/a | 3.12 (0.34) |
| Cystatin-C | -0.16 (0.25) | -0.12 (0.21) | -0.3 (0.25) | -0.16 (0.21) | -0.01 (0.23) |
| UACR | 2.1 (1.1) | 2.01 (1.52) | 2.24 (0.9) | 1.99 (1.06) | n/a |

Biomarker values were natural log transformed and presented as means (standard deviations). Abbreviations: PC, pooled cohort; FHS, Framingham Heart Study; PREVEND, Prevention of Renal and Vascular End-stage Disease; MESA, Multi-Ethnic Study of Atherosclerosis; CHS, Cardiovascular Health Study; PAI-1, plasminogen activator inhibitor-1; sST2, soluble interleukin-1 receptor-like-1; UACR, urinary albumin-to-creatinine ratio.

**Supplemental Table 4. Associations of cardiovascular biomarkers with incident heart failure: men versus women**

|  | Men | | Women | | Interaction |
| --- | --- | --- | --- | --- | --- |
|  | **sHR (95% CI)** | **P-value** | **sHR (95% CI)** | **P-value** | **P_int_** |
| Natriuretic peptides | 1.57 (1.45-1.70) | <0.001 | 1.47 (1.35-1.61) | <0.001 | 0.86 |
| Cardiac troponins | 1.30 (1.22-1.38) | <0.001 | 1.34 (1.26-1.43) | <0.001 | 0.23 |
| PAI-1 | 1.09 (0.95-1.25) | 0.227 | 1.11 (0.93-1.31) | 0.253 | 0.51 |
| D-dimer | 1.17 (1.07-1.27) | <0.001 | 1.24 (1.11-1.38) | <0.001 | 0.24 |
| Fibrinogen | 1.12 (1.05-1.19) | 0.001 | 1.11 (1.03-1.19) | 0.005 | 0.76 |
| C-reactive protein | 1.15 (1.08-1.22) | <0.001 | 1.17 (1.09-1.26) | <0.001 | 0.99 |
| Galectin-3 | 1.04 (0.97-1.11) | 0.284 | 1.13 (1.05-1.22) | 0.001 | 0.04 |
| sST2 | 1.08 (0.99-1.18) | 0.088 | 1.12 (1.02-1.22) | 0.017 | 0.40 |
| Cystatin-C | 1.18 (1.11-1.25) | <0.001 | 1.17 (1.10-1.25) | <0.001 | 0.88 |
| UACR | 1.25 (1.15-1.35) | <0.001 | 1.31 (1.18-1.45) | <0.001 | 0.11 |

Fine-Gray models were adjusted for the competing risk of death, and for the following variables: age, smoking, diabetes mellitus, hypertension, body-mass index, atrial fibrillation, myocardial infarction and presence of left ventricular hypertrophy / left bundle branch block; strata statement included. All biomarkers were natural log-transformed and standardized. Interaction P-value (P_int_) denotes *sex*covariate* interaction on a multiplicative scale in the total population. Abbreviations: CI, confidence interval; PAI-1, plasminogen activator inhibitor-1; sHR, subdistribution hazard ratio per unit change in biomarkers; sST2, interleukin-1 receptor-like 1; UACR, urinary albumin-to-creatinine ratio.

**Supplemental Table 5. Associations of log_2_ transformed biomarkers with incident heart failure:**

**men versus women**

|  | Men | | Women | | Interaction | |  |
| --- | --- | --- | --- | --- | --- | --- | --- |
|  | sHR (95% CI) | P-value | sHR (95% CI) | P-value | | P_int_ | |
| Natriuretic peptides | 1.32 (1.25-1.38) | <0.001 | 1.26 (119-1.33) | <0.001 | | 0.73 | |
| Cardiac troponins | 1.28 (1.19-1.37) | <0.001 | 1.33 (1.25-1.42) | <0.001 | | 0.17 | |
| PAI-1 | 1.08 (0.96-1.23) | 0.20 | 1.09 (0.93-1.27) | 0.29 | | 0.63 | |
| D-dimer | 1.14 (1.06-1.22) | <0.001 | 1.18 (1.08-1.29) | <0.001 | | 0.40 | |
| Fibrinogen | 1.47 (1.17-1.84) | <0.001 | 1.42 (1.11-1.83) | 0.006 | | 0.74 | |
| C-reactive protein | 1.09 (1.05-1.13) | <0.001 | 1.10 (1.05-1.16) | <0.001 | | 0.96 | |
| Galectin-3 | 1.06 (0.92-1.24) | 0.42 | 1.28 (1.09-1.51) | 0.002 | | 0.04 | |
| sST2 | 1.16 (0.98-1.38) | 0.09 | 1.24 (1.04-1.48) | 0.018 | | 0.42 | |
| Cystatin-C | 1.57 (1.30-1.90) | <0.001 | 1.58 (1.29-1.92) | <0.001 | | 0.76 | |
| UACR | 1.15 (1.09-1.20) | <0.001 | 1.17 (1.10-1.25) | <0.001 | | 0.10 | |

To facilitate clinical interpretation, biomarkers were log_2_ transformed for these analyses, and sudistribution hazard ratio (sHR) represents the risk of developing HF per doubling of biomarker. Readers should, however, exercise caution while interpreting sHRs across biomarkers: a 2-fold increment in natriuretic peptides is not the same as a 2-fold increment in cystatin-C. Fine-Gray models were adjusted for the competing risk of death, and for the following variables: age, smoking, diabetes mellitus, hypertension, body-mass index, atrial fibrillation, myocardial infarction and presence of left ventricular hypertrophy / left bundle branch block; strata statement included. Interaction P-value (P_int_) denotes sex*covariate interaction on a multiplicative scale in the total population. Abbreviations: CI, confidence interval; PAI-1, plasminogen activator inhibitor-1; sST2, interleukin-1 receptor-like 1; UACR, urinary albumin-to-creatinine ratio.

**Supplemental Table 6. Cohort-specific associations of biomarkers with incident heart failure**

**in men and in women**

|  | Subdistribution Hazard Ratio (95% Confidence Interval) | | | |
| --- | --- | --- | --- | --- |
| **Biomarkers** | **FHS** | **PREVEND** | **MESA** | **CHS** |
| MEN |  |  |  |  |
| Natriuretic peptides | 1.34 (1.11-1.62) | 1.62 (1.31-2.00) | 2.23 (1.87-2.67) | 1.42 (1.27-1.59) |
| Cardiac troponins | 1.30 (1.12-1.51) | 1.37 (1.16-1.62) | 1.54 (1.36-1.74) | 1.20 (1.10-1.31) |
| D-dimer | 1.13 (0.88-1.44) | n/a | 1.22 (1.06-1.41) | 1.11 (0.99-1.25) |
| Fibrinogen | 1.15 (0.96-1.39) | n/a | 1.21 (1.03-1.42) | 1.08 (1.00-1.17) |
| C-reactive protein | 1.30 (1.07-1.59) | 1.00 (0.78-1.28) | 1.27 (1.07-1.50) | 1.14 (1.05-1.23) |
| Galectin-3 | 1.13 (0.92-1.40) | 1.10 (0.89-1.34) | n/a | 1.02 (0.94-1.11) |
| Cystatin-C | 1.27 (1.05-1.53) | 1.06 (0.91-1.23) | 1.23 (1.11-1.37) | 1.17 (1.07-1.28) |
| UACR | 1.28 (1.01-1.62) | 1.20 (1.04-1.38) | 1.27 (1.13-1.44) | n/a |
| WOMEN |  |  |  |  |
| Natriuretic peptides | 1.43 (1.17-1.76) | 1.96 (1.27-3.03) | 2.47 (1.83-3.32) | 1.36 (1.22-1.52) |
| Cardiac troponins | 1.30 (1.12-1.52) | 1.36 (1.11-1.67) | 1.44 (1.19-1.74) | 1.39 (1.27-1.51) |
| D-dimer | 1.38 (1.04-1.83) | n/a | 1.14 (0.91-1.44) | 1.23 (1.07-1.41) |
| Fibrinogen | 1.32 (1.06-1.64) | n/a | 0.94 (0.77-1.15) | 1.11 (1.02-1.20) |
| C-reactive protein | 1.29 (1.01-1.65) | 1.16 (0.86-1.55) | 1.16 (0.91-1.47) | 1.17 (1.07-1.28) |
| Galectin-3 | 1.29 (1.06-1.56) | 0.92 (0.70-1.21) | n/a | 1.14 (1.05-1.24) |
| Cystatin-C | 1.27 (1.07-1.50) | 1.43 (1.07-1.91) | 1.09 (0.90-1.33) | 1.16 (1.08-1.26) |
| UACR | 1.50 (1.19-1.89) | 1.27 (1.08-1.49) | 1.27 (1.08-1.51) | n/a |

Fine-Gray models were adjusted for the competing risk of death, and for the following variables: age, smoking, diabetes mellitus, hypertension, body-mass index, atrial fibrillation, myocardial infarction, presence of left ventricular hypertrophy / left bundle branch block. All biomarkers were natural log-transformed and standardized. PREVEND models were adjusted for study design. Abbreviation: UACR, urinary albumin-to-creatinine ratio.

All figures and data in the online supplementary material are original, and have not been published in this form before. A full list of participating Multi-Ethnic Study of Atherosclerosis (MESA) investigators and institutions can be found at <https://www.mesa-nhlbi.org>. A full list of principal Cardiovascular Health Study investigators and institutions can be found at <https://chs-nhlbi.org>.
